# A proximal LAVA method for genome-wide association and prediction of traits with mixed inheritance patterns

**DOI:** 10.1186/s12859-021-04436-6

**Published:** 2021-10-26

**Authors:** Patrik Waldmann

**Affiliations:** Research Unit of Mathematical Sciences, University of Oulu, Oulu, Finland

**Keywords:** Machine learning, Regularization, Genetic mapping, Genomic selection

## Abstract

**Background:**

The genetic basis of phenotypic traits is highly variable and usually divided into mono-, oligo- and polygenic inheritance classes. Relatively few traits are known to be monogenic or oligogeneic. The majority of traits are considered to have a polygenic background. To what extent there are mixtures between these classes is unknown. The rapid advancement of genomic techniques makes it possible to directly map large amounts of genomic markers (GWAS) and predict unknown phenotypes (GWP). Most of the multi-marker methods for GWAS and GWP falls into one of two regularization frameworks. The first framework is based on $\ell _1$-norm regularization (e.g. the LASSO) and is suitable for mono- and oligogenic traits, whereas the second framework regularize with the $\ell _2$-norm (e.g. ridge regression; RR) and thereby is favourable for polygenic traits. A general framework for mixed inheritance is lacking.

**Results:**

We have developed a proximal operator algorithm based on the recent LAVA regularization method that jointly performs $\ell _1$- and $\ell _2$-norm regularization. The algorithm is built on the alternating direction method of multipliers and proximal translation mapping (LAVA ADMM). When evaluated on the simulated QTLMAS2010 data, it is shown that the LAVA ADMM together with Bayesian optimization of the regularization parameters provides an efficient approach with lower test prediction mean-squared-error (65.89) than the LASSO (66.11), Ridge regression (83.41) and Elastic net (66.11). For the real pig data the test MSE of the LAVA ADMM is 0.850 compared to the LASSO, RR and EN with 0.875, 0.853 and 0.853, respectively.

**Conclusions:**

This study presents the LAVA ADMM that is capable of joint modelling of monogenic major genetic effects and polygenic minor genetic effects which can be used for both genome-wide assoiciation and prediction purposes. The statistical evaluations based on both simulated and real pig data set shows that the LAVA ADMM has better prediction properies than the LASSO, RR and EN. Julia code for the LAVA ADMM is available at: https://github.com/patwa67/LAVAADMM.

**Supplementary Information:**

The online version contains supplementary material available at 10.1186/s12859-021-04436-6.

## Background

Mendelian, or classical, genetics is the study of traits that is controlled by a single locus. A mutation in a single gene can cause a disease, or another phenotypic alteration, that is inherited according to Mendel’s principles. Those traits are also referred to as monogenic [1]. In humans, there are 5000–8000 monogenic diseases due to mutations in single genes [2], and numerous monogenic diseases can be found also in animals and plants [3, 4]. In contrast, quantitative genetics is generally defined as the study of characters that are influenced by a large number of genes where the effect of each gene is considered to be relatively small [5]. Most diseases and traits of economical importance are considered to have a complex polygenic basis [6]. Oligogenic inheritance refers to an intermediate between monogenic and polygenic inheritance where a trait that is considered to be determined by a small number of genes. Recently, several monogenic diseases have been found to constitue a mixture between effects from one major gene and several mediater genes contributing small effects [7, 8]. For a large part of the twentieth century, quantitative genetics was confined to speculations and restrictive assumptions regarding the effects and distributions of alleles at genetic loci. However, the advent of high-throughput sequencing techniques now makes it possible to assess the direct effects of markers that cover large parts of the genome [9].

In many situations, the genomic data will be wide, i.e. there will be many more predictor variables (*p*) than observations (*n*). Moreover, the predictors are often substantially correlated with each other. Joint modeling of regression coefficients through standard multiple regression is not feasible in these situations. For example, when $p > n$ the ordinary least squares estimator is not unique and will overfit the data with low prediction accuracy as a result. Other problems with wide big data include spurious random correlations, incidental endogeneity, and accumulation of noise [10]. One way to overcome these challenges is to use regularized regression approaches. Ridge regression (RR) [11] estimates the regression coefficients through an $\ell _2$-norm penalized least squares criterion, which means that the coefficients of the predictors are shrunk with the same proportion. However, even though RR can handle correlated predictors, no variables are set to exactly zero and therefore variable selection is not performed. In contrast, the LASSO [12] performs regularization with an $\ell _1$-norm penalty function which shrinks each coefficient by a constant amount $\lambda /2$ (i.e. half of the regularization parameter), and also sets unimportant regression coefficients to exactly zero and therefore performs variable selection. However, the LASSO tends to have problems when predictors are highly correlated or have some form of group structure, and will usually pick one variable and ignore the rest. Simulation studies have shown that neither RR nor the LASSO will universally outcompete the other. In general, one might expect the LASSO to perform better in a setting where a relatively small number of predictors have substantial coefficients, and the remaining predictors have coefficients that are very small or equal zero. RR will achive better prediction accuracy when the response is a function of many predictors, all with coefficients of roughly equal size [13].

Because of the shortcomings of RR and the LASSO, [14] proposed the elastic-net (EN) method, which is based on a penalty that combines the $\ell _1$-norm and $\ell _2$-norm penalties. Hence, the EN can perform variable selection of highly correlated predictors. However, optimization of the elastic-net function involves tuning of two regularization parameters ($\lambda _1$ and $\lambda _2$), or one regularization parameter $\lambda$ and an $\alpha$-ratio that determines how much weight should be given to the LASSO and RR, respectively. [15] demonstrated how cross-validation can be used to find the minimum mean-squared error along a $\lambda$ path for a certain $\alpha$-ratio. [16] suggested 2D-tuning of $\lambda _1$ and $\lambda _2$, but this approach tends to be computationally demanding. Further details on theoretical properties and algorithms for the EN method can be found in [17]. Recently, as an alternative to the EN, [18] developed the LAVA regression model which is based on the splitting of the regression component into one sparse and one dense part. In order to provide identifiability of the separate regression coefficients, the LAVA algorithm relies on the computation of a rather elaborate projection matrix [19].

The LASSO is a specific variant of a structured non-smooth optimization problem, and therefore representative of a more generic class of problems encompassing constrained and nonconvex optimization. In this area, there has been a renewed interest in fast first-order proximal splitting algorithms [20, 21]. The main disadvantage of splitting algorithms is their low speed of convergence since most of them are based on some form of gradient descent approach. Hence, a considerable amount of research effort has been devoted to their tuning and acceleration. [22] proposed the fast iterative shrinkage thresholding algorithm (FISTA), which turns out to be a proximal gradient method for LASSO regularization. A related optimization approach is the alternating direction method of multipliers (ADMM) [23], that easily can be adapted to fast large-scale LASSO regularization of genomic data [24].

The purpose of this study is to develop a proximal ADMM version of the LAVA method and apply it to genomic data where we suspect that the markers follow oligogenetic inheritance. We show how variable splitting in combination with translation mapping provides full identifiability of the regression parameters and results in a computationally efficient approach that can handle the size of typical genome-wide data sets. This is to our knowledge the first implementation of a proximal gradient descent version of the LAVA regularizer. Moreover, the learning rate of the gradient descent iterations is optimized with backtracking line search [20] and the penalty parameters are stochastically tuned with Bayesian optimization using two different acquisition functions [25]. Hence, these optimization procedures provide a considerable computational advancement of hyper-parameter tuning compared to earlier methods that facilitate large scale inference. The statistical properties of the LAVA method is compared with RR, LASSO and EN implementations on a simulated data set intended to mimic oligogenic inheritance and a real data set from pig.

## Results

### Simulated data

After some initial runs with each of the regularizers, it was found that Bayesian optimization (BO) converged faster for the methods with one regularization parameter (i.e. RR and LASSO) when using the upper confidence bound (UCB) acquisition function, and for the methods with two regularization parameters (i.e. EN and LAVA) the mutual information (MI) acquisition function worked better. The lower and upper bounds of $\lambda _1$ were set to 1000.0 and 30,000.0 for RR, and to 10.0 and 2000.0 for the LASSO. The EN bounds were set to 10.0 and 600.0 for $\lambda _1$ and to 0.001 and 1.0 for $\lambda _2$, and for the LAVA they were set to 10.0 and 2000.0 for $\lambda _1$ and to 5000.0 and 300,000.0 for $\lambda _2$. BO was run for 250 iterations for all methods with 4 Gaussian process (GP) function evaluations per iteration. The minimum test MSE was 83.41 and found at $\lambda _1 = 4587.9$ for RR, and for the LASSO, the minimum test MSE was 66.11 at $\lambda _1 = 294.3$ (Table 1). Moreover, the best result for the EN was found at $\lambda _1 = 288.3$ and $\lambda _2 = 0.001$ with a minimum test MSE of 66.11, which means that the EN made no improvements over the LASSO. The best result (MSE = 65.89) of all methods was found for the LAVA at $\lambda _1 = 297.3$ and $\lambda _2 = 211395.0$ (Table 1). Timing of the last evaluation with optimized regularization parameters showed that RR was fastest taking only 10.5 seconds. The LASSO, EN and LAVA were 11.6, 11.7 and 19.2 times slower, respectively (Table 1).

**Table 1:** Minimum test MSE and optimal regularization parameters for RR, LASSO, EN and LAVA evaluated on the simulated QTLMAS data

Method	minMSE	$\lambda _1$	$\lambda _2$	Time
RR	83.41	4587.9		10.5
LASSO	66.11	294.3		121.6
EN	66.11	288.3	0.001	123.2
LAVA	65.89	297.3	211,395.0	201.8

Time in seconds is for the last evaluation with optimized regularization parameters

**Fig. 1:**
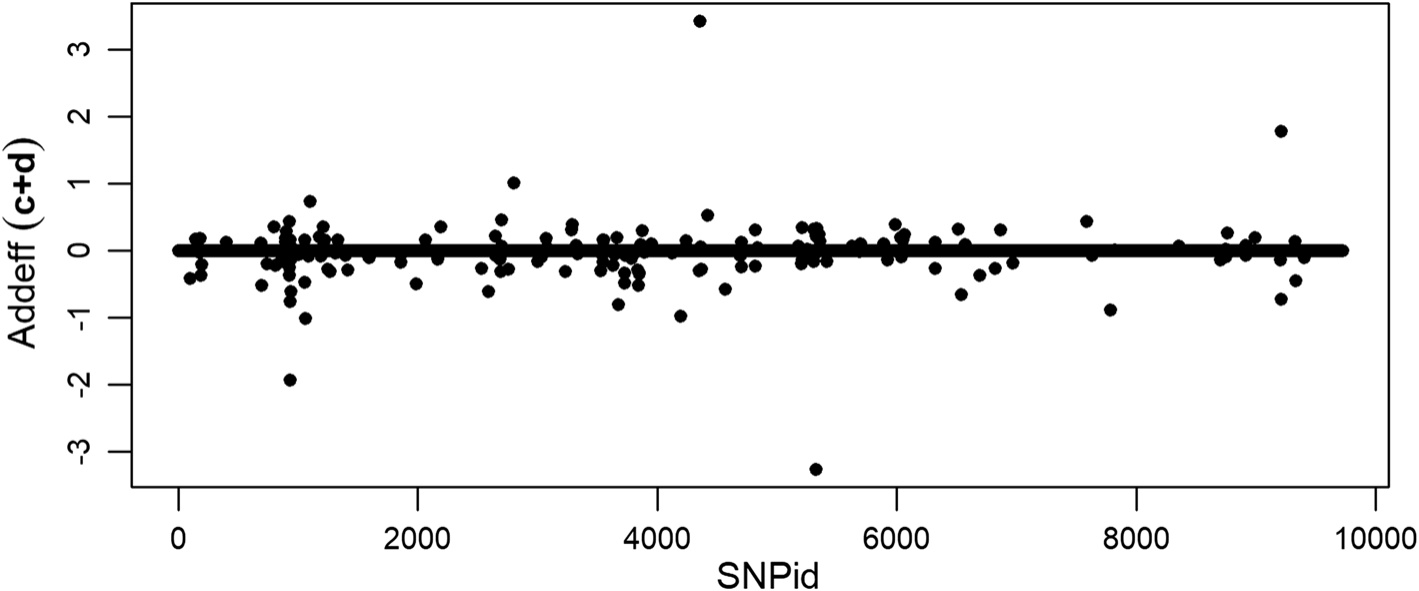
Additive genetic effects from the joint regression coefficients $(c+d)$ of the simulated QTLMAS2010 data

The additive and dominance genetic effects for the LAVA model were also calculated. The additive genetic effects for regression coefficients *c* and *d* were computed as the difference between the regression coefficients of upper homozygote genotype 2 and the lower homozygote genotype 0 for each SNP. Most of the additive effects are captured by the $\ell _1$-norm regularized regression coefficient *c* (Additional file 1), but some additive variation is also explained by the $\ell _2$-norm regularized regression coefficient *d* (Additional file 2). The plot of the joint additive effects $(c+d)$ are dominated by the scale of the $\ell _1$-norm coefficients (Fig. 1).

**Fig. 2:**
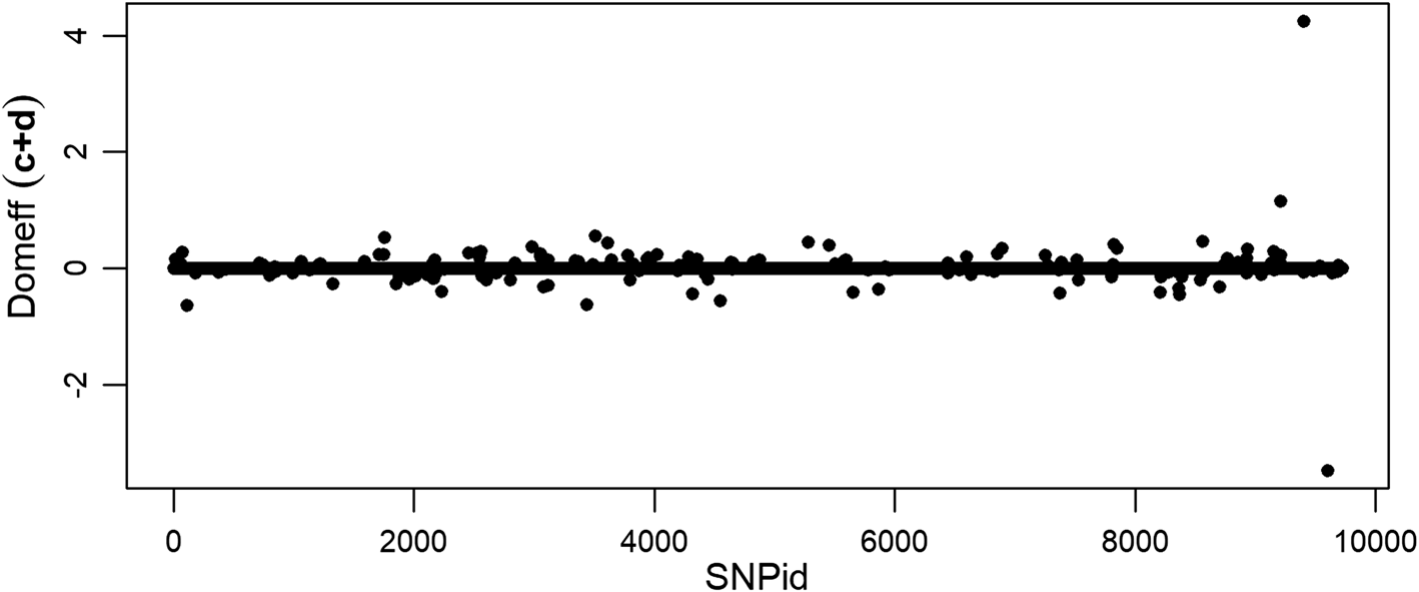
Dominance genetic effects from the joint regression coefficients $(c+d)$ of the simulated QTLMAS2010 data

The dominace genetic effects for regression coefficients *c* and *d* were obtained as the regression coefficient for the heterozygote indicator. It can be seen that the three simulated domianace effects are picked-up well by the $\ell _1$-norm regularized regression coefficient *c* (Additional file 3). The dominance effects of the $\ell _2$-norm regularized regression coefficient *d* are smaller than the domianace effects in *c* (Additional file 4). The joint plot of the dominance components follows the pattern of the additive plot where the the scale of $\ell _1$-norm coefficients dominates (Fig. 2).

### Real data

In the analyzes of the pig data, 5-fold cross-validation with random allocations into training and test data was used to obtain minimum test MSE which was averaged over the folds. We used the same acquisition functions for the pig data as was used for the simulated data. However, the number of iterations was set to 100 and each GP iteration used 3 function evaluations because of the larger number of markers. The lower and upper bounds of $\lambda _1$ were set to 10,000.0 and 250,000.0 for RR, and to 10.0 and 100.0 for the LASSO. For EN, the $\lambda _1$ bounds were choosen to be 10.0 and 200.0, while the bounds of $\lambda _2$ were 5000.0 and 100,000.0. The LAVA bounds of $\lambda _1$ were set to 10.0 and 200.0, and of $\lambda _2$ to 10,000.0 and 200,000.0. The minimum test MSE varied relatively little over the CV-folds for all methods, the largest standard deviation being 0.0435 for RR. The smallest mean minimum test MSE was 0.850 for this data set and encountered with the LAVA method at the average estimates $\lambda _1 = 11.4$ and $\lambda _2 = 44{,}058$ (Table 2). The corresponding minimum test MSE were 0.853, 0.875 and 0.853, for the RR, LASSO and EN methods, respectively (Table 2). The average timing over the folds of the last evaluation with optimized regularization parameters showed that RR and EN were fastest taking 43.4 and 49.4 s, repsctively. The LASSO and LAVA were slower at 229.9 and 336.3 seconds (Table 2).

**Table 2:** Mean minimum test MSE and optimal regularization parameters over 5 CV-folds for RR, LASSO, EN and LAVA evaluated on the pig data

Method	minMSE	$\lambda _1$	$\lambda _2$	Time
RR	0.853	59719		43.4
LASSO	0.875	49.94		229.9
EN	0.853	18.04	9046.7	49.4
LAVA	0.850	53.81	73345	336.3

Time in seconds is the average over the folds for the last evaluation with optimized regularization parameters

**Fig. 3:**
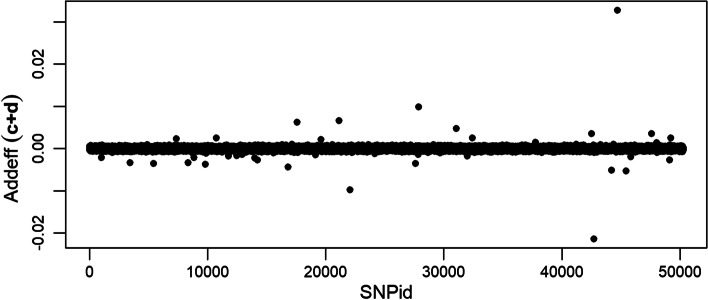
Additive genetic effects from the joint regression coefficients $(c+d)$ of the Cleveland pig data

The additive effects of the $\ell _1$-norm regularized part *c* of the LAVA model are larger in magnitude (Additional file 5) than the additive effects found by the $\ell _2$-norm regularized part *d* (Additional file 6). The plot of joint additive effects $(c+d)$ are dominated by the scale of $\ell _1$-norm coefficients, but the $\ell _2$-norm contribution is proportionally larger than it is for the QTLMAS2010 data (Fig. 3).

**Fig. 4:**
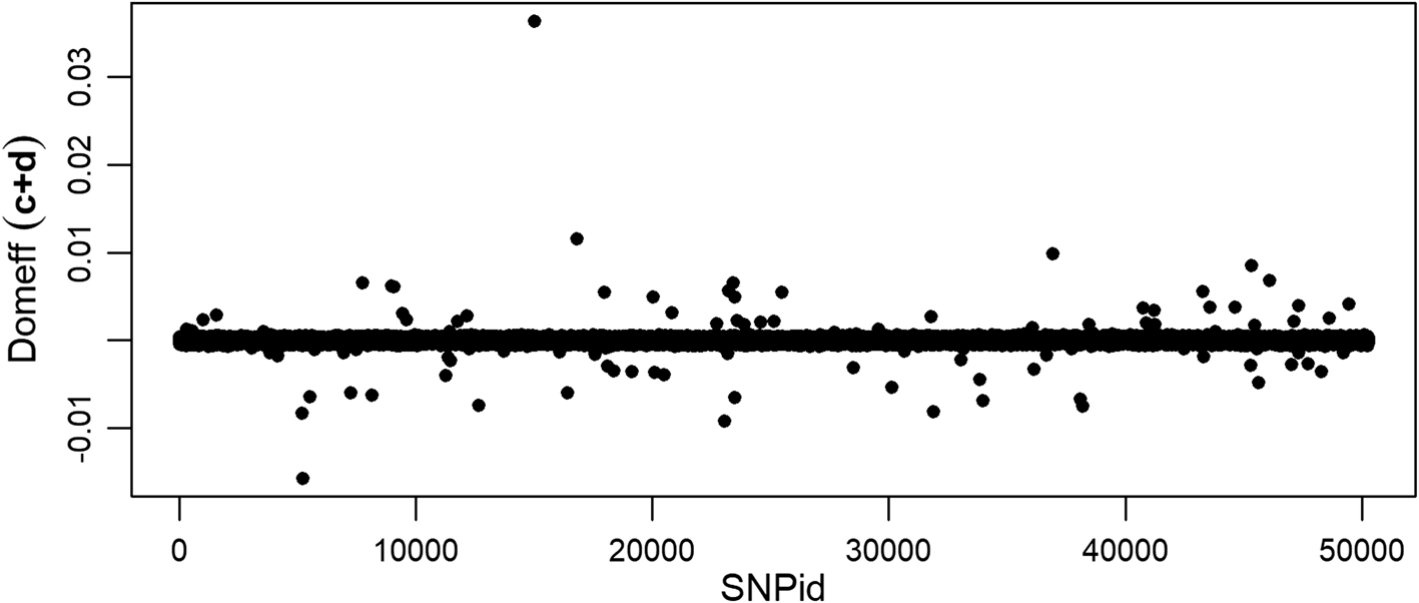
Dominance genetic effects from the joint regression coefficients $(c+d)$ of the Cleveland pig data

A similar result can be seen for the domianace effects where the largest effects are captured for the $\ell _1$-norm part *c* (Additional file 7) and the dominance effects of the $\ell _2$-norm regularized regression coefficient *d* are smaller (Additional file 8). However, also here is the $\ell _2$-norm contribution proportionally larger than what can be seen for QTLMAS2010 data (Fig. 4) . It is worth noting that one major positive additive effect is found at SNP position 44,686 and one major positive dominance effect at SNP position 15,013.

## Discussion

One of the longest standing debates in genetics has been if most quantitative traits are determined by of a few loci with major effects or by many loci with minor effects [26, 27]. Even though it is generally considered that most traits are controlled by a large number of loci with small effects and that this fits well with the infinitesimal model of inheritance [6], it has been stressed that there is plenty of empirical evidence also for traits with major effects loci and the question to answer is not how much does each class contribute but rather ’how do they work together?’ [28]. This discussion is closely intertwined with the statistical methods used for inference of the marker effects. Most methods used for effect estimation are based on linear models with Gaussian likelihood functions and errors, and it can be shown that they fall under the RR framework which means that they implicitly favour the infinitesimal model. On the other side are sparsity inducing methods like the LASSO and Bayesian variable selection with mixture priors that indirectly force the result to one with few loci of major effects. The LAVA method presented in this paper extracts the best of these two worlds and allows for joint estimation of major and minor genetic effects.

The recent focus on sparsity in high-dimensional problems has resulted in a pletora of alternative methods and algorithms [17, 29]. However, it should be emphasized that the joint LAVA estimates $(c+d)$ are dense which puts it in contrast to for example the LASSO and the EN. It has been stressed that the EN should be less sensitive to correlations between predictors than the LASSO because of the RR part in the penalty [17]. However, it is mainly the variable selection properites that are improved with the EN because the prediction error is seldom improved, see for example [30]. [31] also pointed out that the LASSO suffer from unstable selections of correlated variables and inconsistent selections of linearly dependent variables in GWAS data, and put forward the Precision Lasso which promotes sparse variable selection by regularization governed by the covariance and inverse covariance matrices of the explanatory variables. However, they also found that while the variable selection properties improved, there was no improvement in terms of prediction accuracy. These findings contrast with the LAVA method in the current paper which improves in terms of prediction propeties, but puts less focus on the variable selection properties. Initially, we also tried a FISTA version of the LAVA regularizer, but it turned out to be difficult to reach repeatable results with the optimizer. If this was due to implementation issues or general identifiability problems of the $(c+d)$ component is hard to say and requires further investigations.

There has been several comparative studies on the properties of various statistical methods in genome-wide prediction studies. [32] compared eleven genomic prediction methods using wheat, maize and barley data. All prediction models produced similar average prediction accuracies except for SVM. [33] evaluated 14 genomic prediction approaches on 2000 biallelic markers by simulating two complex traits in an F2 and backcross population resulting from crosses of inbred lines. They showed that the parametric methods predicted phenotypic values worse than those of non-parametric models in the presence of epistasis. [34] compared fifteen methods on four datasets (rice, pig, QTLMAS and maize) and found that different methods performed best on different data sets. However, variable selection based approaches (e.g. EN) tended to perform overall better than regularization approaches. [35] compared fourteen prediction methods on simulated data with different genetic architectures and found that when the trait was under additive gene action, the parametric prediction methods outperformed non-parametric ones. On the other hand, when the trait was influenced by epistatic gene action, the non-parametric methods provided more accurate predictions. Hence, the conclusion that can be drawn from these comparative studies is that the prediction properties to a large extent depends on the genetic architecture, which is not surprising since most methods up-to-date either performs favourable on data sets with major gene action or on data with minor polygenic gene action. A large complementary simulation study that evaluates the properties of the LAVA ADMM on different genetic architectures is currently being undertaken.

In regard to these findings, it is interesting to note that the proportion of the variance of the $\ell _2$-norm regularized regression coefficients and $\ell _1$-norm regularized regression coefficients (i.e. $\text{ VAR }(d)/\text{VAR }(c)$) is considerably larger in the real Cleveland pig data (1.24E-3) than in the simulated QTLMAS data (2.55E-6). This measure provides important information regarding the relative importance of minor and major effects and can easily be calulated for each of the norms as long as there are more than one selected marker in the $\ell _1$-norm. Alternatively, one could use $\text{ MEAN }(\text{ ABS }(d))/\text{MEAN }(\text{ ABS }(c))$ in situations where the $\ell _1$-norm component results in only a few selected coefficients of similar size.

## Conclusion

This study presents the LAVA ADMM that is capable of joint modelling of mixtures of monogenic major genetic effects and polygenic minor genetic effects which can be used for both genome-wide assoiciation and prediction purposes. The statistical evaluations based on both a simulated data set and a real pig data set shows that the LAVA ADMM has better prediction properies than the LASSO, RR and EN. However, the LAVA ADMM should be used in combination with these methods because pure sparse major genetic effects architectures are best modelled with the LASSO whereas pure ploygenic minor effects architectures are best modelled with RR.

## Methods and data

### The LAVA regularizer

First, we will review the LAVA regression method [18]. Consider a standard linear regression model

1 $$\begin{aligned} y = Xb+e \end{aligned}$$

where *y* is a response (output) vector of length *n*, *X* is a predictor (input) matrix of size $n \times p$, *b* is a regression coefficient (parameter) vector of length *p* and *e* is a residual (error) vector of length length *n*. Regularization provides a tool to put constraints on the regression coefficients, and a general optimization model can be formulated as

2 $$\begin{aligned} {\hat{b}} = \underset{b}{{\text{argmin}}} \{f(b) + g(b)\} \end{aligned}$$

where *f*(*b*) is a loss function and *g*(*b*) is a penalty function. Ridge regression [11] is obtained as

3 $$\begin{aligned} {\hat{b}} = \underset{b}{{\text{argmin}}} \Vert {y -Xb}\Vert _{2}^{2} + \lambda \Vert {b}\Vert _{2}^{2} \end{aligned}$$

where $\left\| \cdot \right\| _{2}$ is the Euclidean $\ell _2$-norm and $\lambda > 0$ is the penalty parameter. RR produces a dense estimate of *b*. As an alternative, the LASSO [12] can be formulated as

4 $$\begin{aligned} {\hat{b}} = \underset{b}{{\text{argmin}}} \Vert y -Xb \Vert _{2}^{2} + \lambda \Vert b \Vert _{1} \end{aligned}$$

where $\Vert \cdot \Vert _{1}$ is the $\ell _1$-norm. The LASSO performs variable selection and therefore produces a sparse *b* vector, i.e. some entries are set to zero. By combining the $\ell _1$-norm and $\ell _2$-norm penalties we arrive at the elastic-net (EN) method

5 $$\begin{aligned} {\hat{b}} = \underset{b}{{\text{argmin}}} \Vert y -Xb \Vert _{2}^{2} + \lambda _1 \Vert b\Vert _{1}\ + \lambda _2 \Vert b \Vert _{2}^{2} \end{aligned}$$

which has two regularization prameters ($\lambda _1$ and $\lambda _2$) to tune [14].

The LAVA regression model [18] is based on the splitting of the regression component into one sparse and one dense part $b = c+d$, and thereby obtaining the following optimization problem

6 $$\begin{aligned} {\hat{c}},{\hat{d}} = \underset{c,d}{{\text{argmin}}} \Vert y -X(c+d)\Vert _{2}^{2} + \lambda _1 \Vert c \Vert _{1} + \lambda _2 \Vert d \Vert _{2}^{2}, \end{aligned}$$

where the resulting estimator ${\hat{b}} = {\hat{c}}+{\hat{d}}$ is non-sparse. Moreover, they suggested a relatively simple three stage procedure for the estimation of these regression coefficients. At the first stage, define the ridge projection matrix

7 $$\begin{aligned} K_{\lambda _2} = I_n - X(X^{T}X + \lambda _2 I_p)^{-1}X^{T}, \end{aligned}$$

and calculate the transformed response and predictors

8 $$\begin{aligned} {\tilde{y}} = K_{\lambda _2}^{1/2}y, \quad {\tilde{X}} = K_{\lambda _2}^{1/2}X. \end{aligned}$$

The second stage is an ordinary LASSO based on the transformed data

9 $$\begin{aligned} {\hat{c}} = \underset{c}{{\text{argmin}}} \Vert {\tilde{y}} -{\tilde{X}}c \Vert _{2}^{2} + \lambda _1 \Vert c \Vert _{1}, \end{aligned}$$

and the third stage consists of ridge regression on the original data with the sparse LASSO estimator

10 $$\begin{aligned} {\hat{d}} = (X^{T}X + \lambda _2 {I_p})^{-1}X^{T}(y-X{\hat{c}}). \end{aligned}$$

Unfortunately, this approach becomes computationally demanding when the size of *X* gets large.

### Proximal operators

A proximal operator $\text{prox}_f$ is used to evaluate a closed and proper convex function *f*(*u*) of a specific optimization subproblem that is assumed to be easier to solve than the original problem. By iteratively evaluating proximal operators on subproblems, a proximal algorithm converges to the solution of the original problem [36]. The proximal operator is defined as

11 $$\begin{aligned} \text{prox}_f(u) = \underset{v}{{\text{argmin}}} \{f(v)+(1/2)\Vert | v - u \Vert _{2}^{2}\} \end{aligned}$$

where *u* and *v* are vectors of length *p*. The right hand side of the argument is strongly convex so it has a unique minimizer for every $u \in \text{R}^{p}$. A scaled version of (11) is obtained by introducing parameter $\gamma > 0$ resulting in an operator where (1/2) is replaced by $(1/2\gamma )$. This definition indicates that $\text{prox}_f(u)$ is a point that compromises between minimizing *f* and being close to *u*. $\gamma$ can be seen as a trade-off parameter between these two terms. Also note the close relationship between ridge regression and the proximal operator.

The proximal operator has several useful properties [20]. Firstly, for an affine transformation $f(u)=\langle \,z,u\,\rangle + a$ the proximal operator becomes

12 $$\begin{aligned} \begin{aligned} \text{prox}_f(u)&= \underset{v}{{\text{argmin}}} \{\langle \,z,v\,\rangle +a+(1/2)\Vert v -u \Vert _{2}^{2}\}\\&= \underset{v}{{\text{argmin}}} \{\langle \,z,u\,\rangle +a-(1/2)\Vert z \Vert _{2}^{2} + (1/2)\Vert v -(u-z) \Vert _{2}^{2}\}\\&= u-z \end{aligned} \end{aligned}$$

which is a translation mapping. Hence, for a function with a standard addition, it is possible to define a translation function as ${{\mathcal {T}}}(u)=f(u+z)-z$. Another key property is for separable sum functions $f(u,z)=g(u)+h(z)$ where splitting leads to

13 $$\begin{aligned} \text{prox}_f(u,z) = \text{prox}_g(u)+\text{prox}_h(z). \end{aligned}$$

Finally we note that there is a near relationship between proximal operators and gradient descent methods where

14 $$\begin{aligned} \text{prox}_{\gamma f}(u) \approx u-\gamma \nabla f(u) \end{aligned}$$

when $\gamma$ is small and *f*(*u*) is differentiable. In this formulation, $\nabla$ denotes the gradient and $\gamma$ is an equivalent to the learning rate of a gradient optimizer [21].

### LAVA ADMM

Proximal algorithms have become popular for large scale problems in statistics and optimization [21, 36]. Most of them are based on some form of gradient descent approach and a generic iterative algorithm for the optimization problem in (2) follows

15 $$\begin{aligned} \begin{aligned} b^{(k+1)}&= \underset{b}{{\text{argmin}}} \{f(b^{(k)})+\langle \,\nabla f(b^{(k)}),b-b^{(k)}\,\rangle +g(b)+ (1/{2\gamma ^{(k)}})\Vert b -b^{(k)} \Vert _{2}^{2}\}\\&= \text{prox}_{g \gamma }(b^{(k)}-\gamma _{k}\nabla f(b^{(k)})), \end{aligned} \end{aligned}$$

where $\gamma _k$ is the step size and *k* the iteration index.

The alternating direction method of multipliers (ADMM) is an algorithm that solves optimization problems by dividing them into smaller subproblems, each of which are then easier to manage. This feature is very advantageous for a broad spectrum of applications and therefore it has become a benchmark method. First, consider for problem (2) that

16 $$\begin{aligned} \begin{aligned} {\hat{b}} = \underset{b}{{\text{argmin}}} \{f(b) + g(b)\}\quad \iff \quad {\hat{b}} =&\underset{b}{{\text{argmin}}} \{f(b) + g(u)\}\\&\text{subject to}\quad b=u \end{aligned} \end{aligned}$$

then, by combining the augmented Lagrangian

17 $$\begin{aligned} \begin{aligned} L_{\gamma }(b,u,z) = f(b)+g(u)+z^{T}(Xb -Xu-y)+(\gamma /2)\Vert Xb-Xu -y \Vert _{2}^{2}, \end{aligned} \end{aligned}$$

with the method of multipliers we end up with an iterative scheme for ADMM according to

18 $$\begin{aligned} \begin{aligned} b^{(k+1)}&=\underset{b}{{\text{argmin}}} L_{\gamma }(b^{(k)},u^{(k)},z^{(k)})\\ u^{(k+1)}&=\underset{u}{{\text{argmin}}} L_{\gamma }(b^{(k+1)},u^{(k)},z^{(k)})\\ z^{(k+1)}&= z^{(k)}+\gamma (Xb^{(k+1)}-Xu^{(k+1)}-y), \end{aligned} \end{aligned}$$

which can be reformulated using proximal operators as

19 $$\begin{aligned} \begin{aligned} b^{(k+1)}&= {\text{prox}}_{f\gamma }(u^{(k)}-z^{(k)})\\ u^{(k+1)}&= {\text{prox}}_{g\gamma }(b^{(k+1)}+z^{(k)})\\ z^{(k+1)}&= z^{(k)}+b^{(k+1)}-u^{(k+1)}. \end{aligned} \end{aligned}$$

It is now straightforward to implement a LAVA ADMM by first defining two translation functions ${{\mathcal {T}}}(u)=f(u+v)-v$ and ${{\mathcal {T}}}(v)=f(v+u)-u$, and then iterating

20 $$\begin{aligned} \begin{aligned} c^{(k+1)}&= {\text{prox}}_{{\mathcal {T}}(u)\gamma }(u^{(k)}-z^{(k)})\\ u^{(k+1)}&= {\text{prox}}_{g\gamma }(c^{(k+1)}+z^{(k)})\\ z^{(k+1)}&= z^{(k)}+c^{(k+1)}-u^{(k+1)}\\ d^{(k+1)}&= {\text{prox}}_{{\mathcal {T}}(v)\delta }(v^{(k)}-w^{(k)})\\ v^{(k+1)}&= {\text{prox}}_{h\delta }(d^{(k+1)}+w^{(k)})\\ w^{(k+1)}&= w^{(k)}+d^{(k+1)}-v^{(k+1)} \end{aligned} \end{aligned}$$

where $\text{prox}_{g\gamma }()$ is the soft-thresholding function with learning rate $\gamma$ defined as

21 $$\begin{aligned} \begin{aligned} \text{prox}_{g\gamma }(c+z)={\mathcal {S}}_{\gamma }(c+z)=[|c+z|-\gamma ]_{+}\text{sgn}(c+z), \end{aligned} \end{aligned}$$

and $\text{prox}_{h\delta }()$ is the $\ell _2$-norm regularization function with learning rate $\delta$. The iterations are terminated when convergence is reached according to $\Vert (c^{(k)}+d^{(k)})-(u^{(k)}+v^{(k)})\Vert _{\infty } \le \beta (1+\Vert z^{(k)}+w^{(k)})\Vert _{\infty })$ for tolerance parameter $\beta$ which was set to $10^{-5}$.

There are two main approaches to determine the learning rate $\gamma$ and $\delta$ [20]. Firstly, since *f*(*b*) is convex, and therefore also Lipschitz continuous with inequality $|f(b)-f(b_0)|\le L \left\| b-b_0\right\|$, the Lipschitz constant can be calculated as $L=\lambda _{\text{max}}(X^{T}X)$ where $\lambda _{\text{max}}$ denotes the maximum eigenvalue. A constant step size for all *k* can be chosen as $\gamma ^{k}=1/L$. Unfortunately, the computation of the eigenvalues becomes labor-some when the size of *X* reaches an order of around $10^{4}$. The second option is to use backtracking line-search which can be implemented for $\gamma$ following

22 $$\begin{aligned}&\text{Set} \quad \alpha = 0.5, \quad \gamma ^{(k=2)}=0.9\\&\text{For each iteration}\quad k \\&\gamma ^{(k)} = \gamma ^{(k-1)} \\&\text{while}\qquad f(u^{(k)})> \{f(c^{(k)})+\\&\qquad \qquad \gamma ^{(k)}\nabla f(c^{(k)})^{T}(-c^{(k)})+\\&\qquad \qquad (1/2\gamma ^{(k)})\left\| -(c^{(k)}\right\| ^{2}_{2}\} \\&\qquad \qquad \text{repeat}\quad \gamma ^{(k)} = \alpha \gamma ^{(k)}\\&\text{end} \end{aligned}$$

where $\nabla f(c^{(k)})=X^{T}(X(c^{(k)})-y)$ is the gradient. The same procedure is applied to $\delta$ by replacing $c^{(k)}$ and $u^{(k)}$ with $d^{(k)}$ and $v^{(k)}$, respectively.

### Bayesian optimization of the penalty parameters

Tuning of the penalty parameters $\lambda _1$ and $\lambda _2$ can be performed with cross-validation and grid search, but the number of evaluations easily becomes very large. For example, 100 values per penalty parameter amounts to optimizing 10,000 models per fold. Bayesian optimization (BO) is a sequential approach for global optimization that has become popular for tuning of hyperparameters in machine learning [37]. In BO, the objective function $l(\mathbf {\lambda })$ is evaluated at *T* sequential points $\text{MSE}^{(1)} = l(\lambda ^{(1)}),\text{MSE}^{(2)} = l(\lambda ^{(2)}),\ldots ,\text{MSE}^{(T)} = l(\lambda ^{(T)})$, where $\text{MSE}$ is the negative test mean squared error and the penalty parameters are collected in a vector $\mathbf {\lambda }=[\lambda _1,\lambda _2]$. By assuming that the negative test mean squared error follows a Gaussian distribution

23 $$\begin{aligned} \text{MSE}^{(t)} \sim N(l(\lambda ^{(t)}),\sigma ^{2}) \end{aligned}$$

and assigning a Gaussian process prior over the objective function

24 $$\begin{aligned} l(\lambda ) \sim \mathcal {GP}(m(\lambda ),k(\lambda ,\lambda ')) \end{aligned}$$

where the mean function $m(\lambda )$ usually is set to zero and the covariance function (i.e. kernel) needs to be chosen, the posterior distribution will be

25 $$\begin{aligned} l |\ \lambda \sim N(K_{ll}(K_{ll}+\sigma ^{2}I)^{-1}(K_{ll}-K_{ll}(K_{ll}+\sigma ^{2}I)^{-1}K_{ll}), \end{aligned}$$

where $K_{ll}=k(\lambda ,\lambda )$. Given that the likelihood, the posterior and the conditional distribution of future observations all are Gaussian, the predictive distribution for $\text{MSE}^{(t+1)}$ will also be Gaussian

26 $$\begin{aligned} \text{MSE}^{(t+1)}\ |\ \lambda ^{(t+1)},\sigma ^{2} \sim N(\mu (\lambda ^{(t+1)}),\Sigma (\lambda ^{(t+1)},\lambda ^{(1,\ldots ,t)})+\sigma ^{2}I), \end{aligned}$$

where $$\mu (\lambda ^{(t+1)})=k(\lambda ^{(t+1)},\lambda )(K_{ll}+\sigma ^{2}I)^{-1}\text{MSE}$$ and $$\Sigma (\lambda ^{(t+1)},\lambda ^{(1,\ldots ,t)})=k(\lambda ^{(t+1)},\lambda ^{(t+1)})-(K_{ll}+\sigma ^{2}I)^{-1}k(\lambda ^{(1,\ldots ,t)},\lambda ^{(t+1)}).$$

The main idea behind BO is to perform a proxy optimization based on an acquisition function to determine the new prediction points of $\lambda$ to evaluate in the next iteration following

27 $$\begin{aligned} \lambda ^{(t+1)} = \underset{\lambda }{{\text{argmax}}} \{\psi (\lambda ) + \phi (\lambda )\}, \end{aligned}$$

where $\psi (\lambda )$ is driven by the mean function $\mu (\lambda )$ and determines the exploitation ability, whereas $\phi (\lambda )$ is determined by the variance function $\Sigma (\lambda )$ and controls the amount of exploration. There are several acquisition functions that trade-off between exploitation and exploration in different ways [25]. [38] introduced the Gaussian process upper confidence bound (GP-UCB)

28 $$\begin{aligned} \lambda ^{(t+1)} = \underset{\lambda }{{\text{argmax}}} \{\psi (\lambda ) + \beta \phi (\lambda )\}, \end{aligned}$$

where $\beta$ is a tuning parameter that determines the trade-off between exploitation and exploration. [39] recommended to use the mutual information (GP-MI) acquisition function

29 $$\begin{aligned} \lambda ^{(t+1)} = \underset{\lambda }{{\text{argmax}}} \{\mu (\lambda ^{(t)}) + \sqrt{\nu }(\sqrt{\Sigma (\lambda ^{(t)})+\xi ^{(t-1)}}-\sqrt{\xi ^{(t-1)}})\}, \end{aligned}$$

where $\nu = \text{log}(2/\delta )$ is a calibration parameter that needs to be chosen for confidence $0<\delta <1$ (in practice values between $10^{-1}$ and $10^{-9}$ seems to have similar effect). The parameter $\xi$ controls the amount of exploration and is calculated based on the mutual information $I(\lambda ^{(1,...t)})=(1/2)\text{log}\ \text{det}(I+\sigma ^{-2}K_{ll})$ following $\xi ^{(t)}={{\text{max}}{I(\lambda ^{(1,...t)})}}$. Hence, the amount of exploration increases with *t*. To reach convergence of the BO (i.e. no more decrease in test MSE), it is recommended to evaluate different parameter bounds and different acquisition functions for different data sets.

### Implementation

The LAVA ADMM algorithm was implemented in Julia 1.5 [40] using the ProximalOperators package [41]. The data sets were analyzed with RR, LASSO, EN and LAVA implementations using the ADMM algorithm. The BO was performed with the BayesianOptimization package with an ElasticGPE model that avoids refitting of the whole Gaussian process and the squared exponential automatic relevance determination (SEArd) kernel [42]. The initial values of ${\hat{b}}$, ${\hat{c}}$ and ${\hat{d}}$ were set to the marginal covariances between *y* and *X* multiplied by 0.0001. All analyses were performed with a Lenovo ThinkPad laptop with Intel Core i5-8265U 16GB RAM and Windows 10.

### Simulated data

The simulated data encompass 3226 individuals organised in a 5 generation pedigree originally created for the QTLMAS2010 work-shop [43]. 20 individuals (5 males and 15 females) act as founders of the pedigree, and by mating each female once they give birth to approximately 30 progeny. A neutral coalescent model was used to simulate the SNP data where the genome is made up of five autosomal chromosomes each with a length of 100 Mbp. The procedure resulted in 10,031 markers, where 263 SNPs became monomorphic and 9768 SNPs turned out to be biallelic.

The continuous quantitative trait is controlled by 9 major QTLs at fixed positions, including two pairs of epistatic genes, 3 maternally imprinted genes and two additive major genes with phenotypic effects of − 3 and 3. The additive genes are positioned at SNP indices 4354 and 5327, whereas the major epistatic locus is at SNP 931. Moreover, 28 minor QTLs, randomly dispersed on chromosome 1–4, have their additive effects sampled from a truncated normal distribution and their effects vary between − 1.98 and 1.93. The QTLs are enclosed by 19 to 47 polymorphic SNPs located within 1 Mb distance from the QTLs. A total of 364 SNPs exhibit moderate to high linkage disequilibrium (LD) with the QTLs. Hence, the trait can be considered to be an example of oligogenic inheritance because it is controlled by both a few major QTLs and a larger number of minor QTLs. However, the true number and positions of the minor QTLs are unknown due to the random sampling of these QTL effects.

In addition, one dominance locus was positioned at SNP number 9212 by allocating an effect of 5.00 to the heterozygote and a value of 5.01 to the upper homozygote. Furthermore, one over-dominance locus was placed at SNP 9404 by assigning an effect of 5.00 to the heterozygote, and an effect of − 0.01 to the lower homozygote and 0.01 to the upper homozygote. Lastly, by assigning a value of − 5.00 to the heterozygote, an effect of − 0.01 to the lower homozygote and 0.01 to the upper homozygote, one under-dominance locus was created at SNP id 9602. The effects of these new dominance QTLs were added to the original phenotype values. SNPs with minor allele frequency (MAF) less than 0.01 was discarded which ended up in 9723 markers. These SNPs were transformed into one-hot encoding which means one indicator variable for each genotype. Hence, the final number of genomic markers was 29169. Generation 1 to 4 (individual 1 to 2326) were used as training data and generation 5 (individual 2327 to 3226) acted as test data.

### Real data

In order to evaluate the methods on a typical real data set, we used a public pig dataset containing 3534 individuals with high-density genotypes, phenotypes, and estimated breeding values for five anonymous traits [44]. Genotypes were scored using the PorcineSNP60 chip, and after quality control, 52,842 SNPs remained. Missing SNPs with both known and unknown positions were imputed using a probability score. The data was anonymised by randomising the map order and recoding the SNP identities. The number of SNPs was further reduced in this study using a more stringent $\text{MAF}<0.01$, which resulted in a final number of 50,276 SNPs.

Most of the genotyped animals were measured for five purebred traits (phenotypes in a single nucleus line). Heritabilities ranged from 0.07 to 0.62. For this study, we chose the trait that had a heritability of 0.38. The phenotypic data points were adjusted for environmental factors and rescaled by correcting for the overall mean. By discarding individuals with missing phenotype data a final number of 3141 individuals was obtained.

## Supplementary Information

**Additional file 1.** **Supplementary 1.** Additive genetic effects from the $\ell _1$-norm regression coefficients *c* of the simulated QTLMAS2010 data

**Additional file 2.** **Supplementary 2.** Additive genetic effects from the $\ell _2$-norm regression coefficients *d* of the simulated QTLMAS2010 data.

**Additional file 3.** **Supplementary 3.** Dominance genetic effects from the $\ell _1$-norm regression coefficients *c* of the simulated QTLMAS2010 data.

**Additional file 4.** **Supplementary 4.** Dominance genetic effects from the $\ell _2$-norm regression coefficients *d* of the simulated QTLMAS2010 data.

**Additional file 5.** **Supplementary 5.** Additive genetic effects from the $\ell _1$-norm regression coefficients *c* of the Cleveland pig data.

**Additional file 6.** **Supplementary 6.** Additive genetic effects from the $\ell _2$-norm regression coefficients *d* of the Cleveland pig data.

**Additional file 7.** **Supplementary 7.** Dominance genetic effects from the $\ell _1$-norm regression coefficients *c* of the Cleveland pig data.

**Additional file 8.** **Supplementary 8.** Dominance genetic effects from the $\ell _2$-norm regression coefficients *d* of the Cleveland pig data.

## Data Availability

The simulated QTLMAS2010 data and Julia code for the LAVA ADMM is available at: https://github.com/patwa67/LAVAADMM. The real pig data is available at: https://www.g3journal.org/content/2/4/429.supplemental.
