# Supplementary material for: A proximal LAVA method for genome-wide association and prediction of traits with mixed inheritance patterns
Source: BMC Bioinformatics. 2021 Oct 26;22:523. doi: 10.1186/s12859-021-04436-6 (PMC8547073; doi:10.1186/s12859-021-04436-6)
Supplement: Supplementary file 1 — Additional file 1. Supplementary 1. Additive genetic effects from the $\ell _1$ℓ1-norm regression coefficients c of the simulated QTLMAS2010 data [file 12859_2021_4436_MOESM1_ESM.docx]

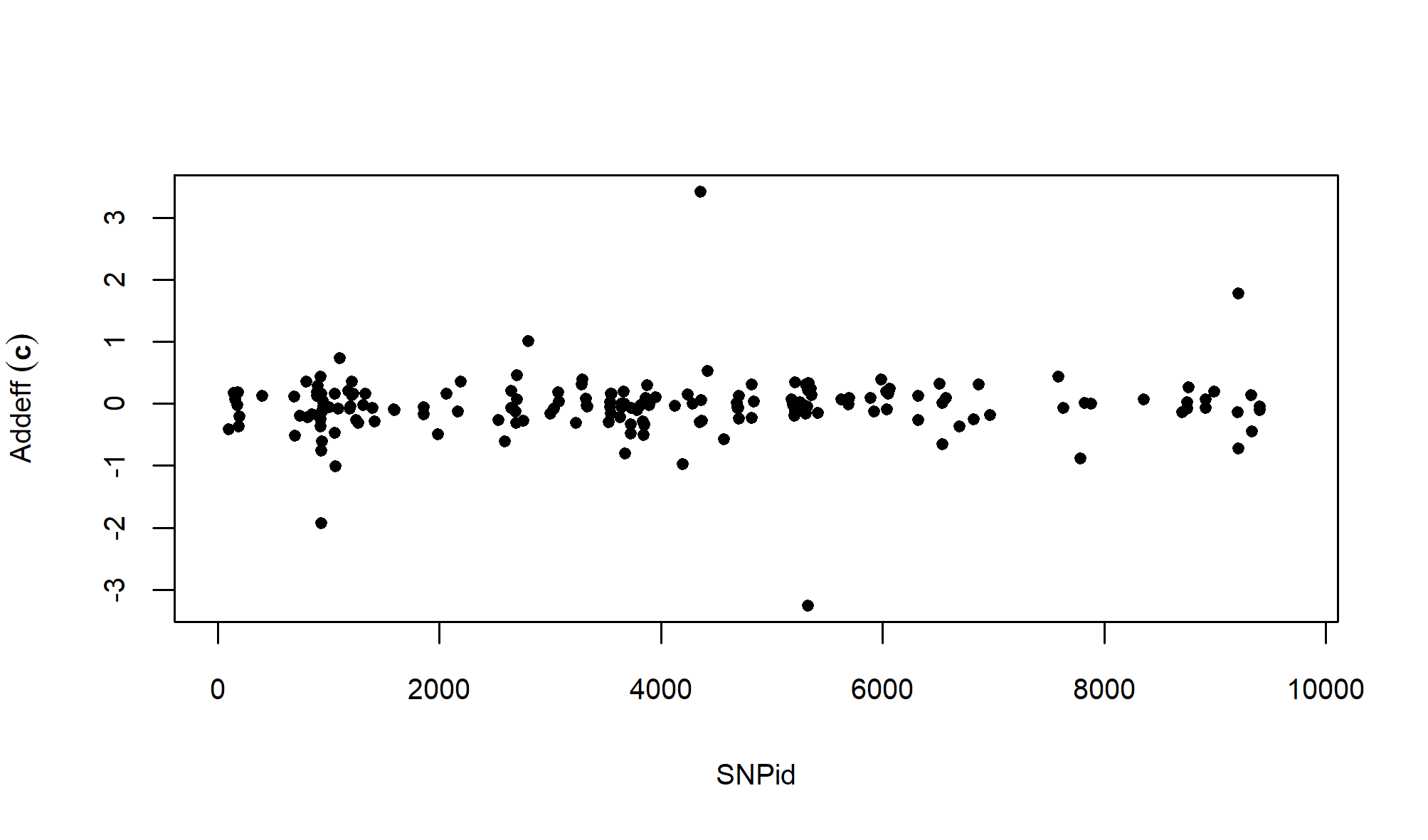


**Supplementary 1.** Additive genetic effects from the $\mathcal{l}_{1}$-norm regression coefficients *c* of the simulated QTLMAS2010 data.
