# Supplementary material for: A proximal LAVA method for genome-wide association and prediction of traits with mixed inheritance patterns
Source: BMC Bioinformatics. 2021 Oct 26;22:523. doi: 10.1186/s12859-021-04436-6 (PMC8547073; doi:10.1186/s12859-021-04436-6)
Supplement: Supplementary file 7 — Additional file 7. Supplementary 7. Dominance genetic effects from the $\ell _1$ℓ1-norm regression coefficients c of the Cleveland pig data. [file 12859_2021_4436_MOESM7_ESM.docx]

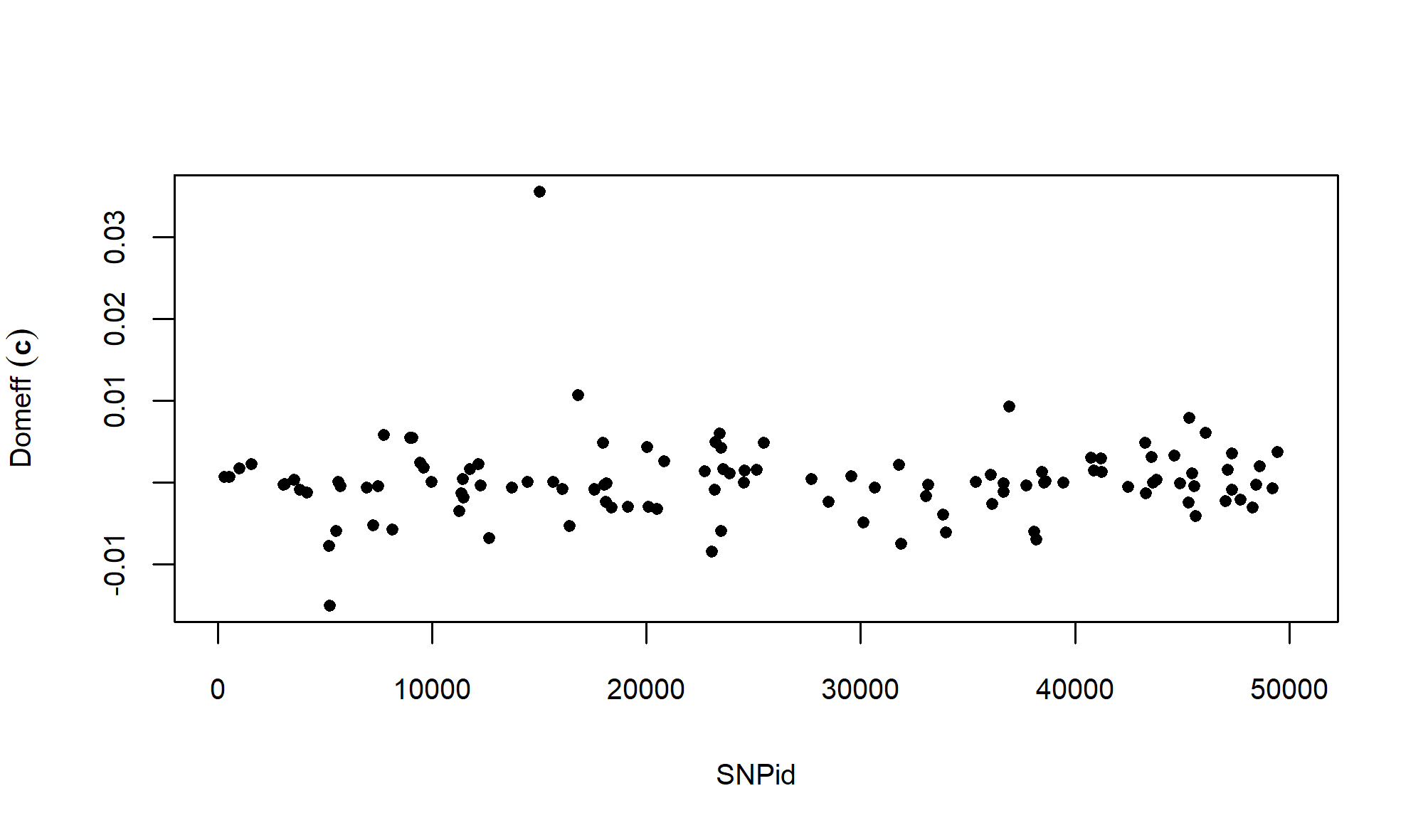


**Supplementary 7.** Dominance genetic effects from the $\mathcal{l}_{1}$-norm regression coefficients *c* of the Cleveland pig data.
